# Controlling false positive rates in mass-multivariate tests for electromagnetic responses

**DOI:** 10.1016/j.neuroimage.2011.02.072

**Published:** 2011-06-01

**Authors:** Gareth R. Barnes, Vladimir Litvak, Matt J. Brookes, Karl J. Friston

**Affiliations:** a The Wellcome Trust Centre for Neuroimaging, Institute of Neurology, UCL, 12 Queen Square, London, WC1N 3BG, UK; b Sir Peter Mansfield Magnetic Resonance Centre, School of Physics and Astronomy, University of Nottingham, University Park, Nottingham, NG7 2RD, UK

## Abstract

We address the problem of controlling false positive rates in mass-multivariate tests for electromagnetic responses in compact regions of source space. We show that mass-univariate thresholds based on sensor level multivariate thresholds (approximated using Roy's union–intersection principle) are unduly conservative. We then consider a Bonferroni correction for source level tests based on the number of unique lead-field extrema. For a given source space, the sensor indices corresponding to the maxima and minima (for each dipolar lead field) are listed, and the number of unique extrema is given by the number of unique pairs in this list. Using a multivariate beamformer formulation, we validate this heuristic against empirical permutation thresholds for mass-univariate and mass-multivariate tests (of induced and evoked responses) for a variety of source spaces, using simulated and real data. We also show that the same approximations hold when dealing with a cortical manifold (rather than a volume) and for mass-multivariate minimum norm solutions. We demonstrate that the mass-multivariate framework is not restricted to tests on a single contrast of effects (cf, Roy's maximum root) but also accommodates multivariate effects (cf, Wilk's lambda).

## Research highlights

► We aim to estimate the number of independent tests made in MEG source space. ► We trial a heuristic based on the number of unique lead field extrema. ► We compare Bonferroni corrected tests using this heuristic to permutation methods. ► The heuristic performs well for both mass-univariate and mass-multivariate tests.

## Introduction

Electromagnetic (M/EEG) signals are information rich; a simple task will elicit not only a poly-phasic evoked response but a complex pattern of time-evolving event-related spectral power changes (Pfurtscheller and Lopes da Silva, 1999). Such signals are difficult to capture as a univariate response, as this necessitates the selection of a single feature of interest (for example the average power decrease in a particular frequency band over a pre-specified time window). Here, we consider the problem of controlling false positive rates in mass-univariate and mass-multivariate statistical parametric maps (SPMs).

The question of whether to treat EEG or MEG responses at a particular point in (sensor or source) space as a multivariate response or a univariate response over an extra (e.g., time or frequency) dimension of the statistical search space has a long history (see (Kiebel and Friston, 2004) for discussion). Recent work (Soto et al., 2009) has pursued the use of mass-multivariate tests to describe experimental effects across MEG source space. Instead of testing for a univariate response at each source location (e.g. amplitude or power), one can exploit the high temporal resolution of M/EEG to apply a multivariate test over a number time bins (or frequencies): In other words, treat the source activity as a multivariate response over time (or frequencies). Mass-multivariate testing in MEG offers many exciting possibilities, allowing one to capture the high dimensional aspect of electromagnetic responses, like subtle stimulus-dependent changes in spectral responses (Duncan et al., 2010; Swettenham et al., 2009). Furthermore, this approach finesses the multiple comparisons problem inherent in multiple univariate tests; e.g., tests for responses in different frequency bands (Singh et al., 2003).

Here, we propose a simple heuristic to estimate the global threshold controlling the false positive rate of mass-multivariate tests in statistical parametric maps. To date such thresholds have been usually derived through permutation tests (Pantazis et al., 2003, 2005; Singh et al., 2003; Soto et al., 2009). One notable exception is the proposal to use a sensor level multivariate threshold to control source level mass-univariate false positive rates (Carbonell et al., 2004). The intuition behind this work is based on Roy's union intersection theorem, which essentially says that one can express the multivariate test of sensor data as a set of univariate tests, over all possible linear combinations of sensors (i.e., putative sources). If a combination (source) exists, for which the null can be rejected under any univariate test, then one can reject the multivariate null. The nice thing about this is that treating the M/EEG data-features as *n* observations over *m* sensors, enables one to control the false positive rate of the sensor level tests using analytical approximations to well known distributions (e.g., Hotelling's T square or Roy's maximum root). In other words, by rejecting the multivariate null at the sensor level one is also rejecting the null on one or more univariate tests on linear combinations of sensors (or sources). This means the same correction can be applied to both sensor level and source level data. Our attention was drawn to this approach, which we shall refer to as the multivariate sensor (MVS) method, because it can easily be extended to threshold mass-multivariate SPMs by simply increasing the effective number of sensors (e.g., 3 × *m* for 3 temporal features per sensor). In this work, we evaluate the sensitivity of this approach and find it wanting. We consider an alternative approach based on Bonferroni correcting source level tests for the number of effective (i.e., independent) sources.

The paper begins with the formulation of a mass-multivariate beamformer SPM comprising a Chi squared statistic (based on Wilk's lambda) at each source location. To control family wise error rate over sources, we need to control the false positive rate of the maximum of these statistics over a pre-specified search region of source space. In the first section, we deal with the mass-univariate case and show that the MVS threshold is conservative with respect to source level permutation tests. Using the source level permutation distribution as (an assumption free) ground truth, we then try to establish how well these thresholds can be approximated by Bonferroni corrections based on the number of independent sources, as assessed with simple heuristics. In other words, we consider the number of independent sources as the number that provides exact control over false positive rates, under a Bonferroni correction. This provides a reference against which to evaluate different (heuristic) estimates of the number of effective sources. We show the heuristic provides a reasonable estimate of the permutation threshold for a variety of source spaces and regions of interest. We show that the same approach can be extended to deal with mass-multivariate tests, where more than a single data feature is tested at each source location. We also show that the approximation holds for mass-multivariate minimum norm solutions constrained to a cortical manifold. Importantly, the same approximations hold for mass-multivariate tests of univariate and multivariate contrasts of effects. Finally, we apply the method to a real data example to demonstrate the utility of a framework that accommodates multivariate effects.

## Methods

There are two sorts of multivariate statistical tests considered in this section: We will consider sensor-level multivariate tests, in which each MEG channel at a specific time (or frequency) bin is treated as a data feature. In other words, the multivariate observation is over sensors. We also consider source-level multivariate tests in which the features are time (or frequency) bins at each source location (i.e., mass-multivariate tests). In the limit, of a single source level-feature (as shown in Fig. 2) this reduces to mass-univariate tests over all sources. In this setting, mass univariate tests in source space can be formulated as multivariate tests in sensor space (by virtue of the linear mapping between centres and sources (Carbonell et al., 2004)). Our primary interest is in controlling false-positive rates for mass-multivariate tests in source space. However, we introduce multivariate sensor-level tests because they can be used to provide control over mass-univariate source tests.

### Mass-multivariate source level tests

Let the data $B_{t}\in C^{m\times k}$ comprise *k* complex valued Fourier coefficients describing the signal on *m* MEG sensors at trial *t*, where *t* ∈ 1, …, *n*(one could equally work well in the time domain but for tests on the power spectrum it is more efficient to compute a single Fourier transform rather than compute a new transform at each source). Let the source space consist of *p* dipolar sources each at separate locations *i*. The design matrix $X\in R^{n\times q}$ tries to explain *n* (trials) rows with *q* columns encoding experimental effects and confounds (e.g., breathing artefacts). A contrast matrix $c\in R^{q\times u}$ describes the *u* linear combinations of columns to test against the null. Unless otherwise stated, we will use a 3 column design matrix, with two columns indicating stimulus on (resp. off) and a third column of ones representing any DC confound. To compare the stimulus on and off periods, controlling for the confound, the contrast matrix would be *c* = [1, − 1, 0]^*T*^. The effects of interest are given by

(1) $$X_{h}=Xc\text{.}$$

Prior to the source level computations one can remove confounds from the data and design

(2) $$X_{0}=X-X_{h}c^{+}$$

where *X*_0_ spans the space orthogonal to Xc and the + operator is the Moore–Penrose pseudo inverse.

The following describes a standard scalar LCMV beamformer implementation (Barnes and Hillebrand, 2003; Brookes et al., 2005, 2008; Robinson and Vrba, 1998; Sekihara et al., 2004). The weight vectors w_i_ for multivariate beamforming are constructed at each location *i* (Van Veen et al., 1997) with a regularisation parameter of zero (Barnes and Hillebrand, 2003).

(3) $$W_{i}=\frac{L_{i}C^{-1}}{\left(L_{i}C^{-1}L_{i}^{T}\right)}$$

where $C\in R^{m\times m}$ is the data covariance matrix and L_i_ is the *i*-th row of the lead field matrix $L\in R^{p\times m}$ of length *m* at location *i*. For grids where no source orientation is specified the optimal direction can be selected through the method of Sekihara et al. (2004). For cortical meshes where the orientation is known, there is a single possible W_*i*_. Alternatively for minimum norm solutions, we make a single computation of W for all source elements based on

(4) $$W=L^{+}\text{.}$$

At each source location let the activity over multiple trials be given by

(5) $$Y=\left[\begin{array}{c} y_{1}\\ \vdots \\ y_{n} \end{array}\right]$$

where each row of *y*_t_ corresponds to a vector of complex Fourier coefficients for trial *t* at location *i*. The power for trial *t* at frequency *j* is simply

(6) $$y_{\mathrm{tj}}=(W_{i}B_{\mathrm{tj}}){\left(W_{i}B_{\mathrm{tj}}\right)}^{*}$$

where * is the complex conjugate operator and *j* = 1 to *k*. For tests on the evoked response we can split the sine and cosine components of the complex Fourier transform into separate columns of Y so that

(7) $$y_{\mathrm{tj}}=g(W_{i}B_{\mathrm{tj}})$$

where *g* is an operator that returns the real parts of the Fourier transform for *j* = 1 to *k*, and imaginary parts for *j* = *k* + 1 to 2*k*. In order to directly compare statistics on the evoked 2*k* and induced *k* responses. In this paper, we used only the real evoked Fourier components.

These transformations provide the data features, where $Y\in R^{n\times k}$ is the power or $Y\in R^{n\times 2k}$ is the amplitude matrix of frequencies at cortical location *i*. We now remove the confounds from the data and reduce its dimensionality using an eigen decomposition:

(8) $$\begin{array}{l} Y_{v}=Y_{h}U_{v}\\ Y_{h}=Y-X_{0}(X_{0}^{+}Y)\\ USU^{T}=Y_{h}^{T}Y_{h}\text{.} \end{array}$$

So that $Y_{v}\in R^{n\times v}$ *Y* contains the first *v* principal components of the power spectrum (or complex amplitude spectrum) spanned by the principal eigenvectors $U_{v}\in R^{k\times v}$. We now model these reduced data features with a general linear model (where ε is a noise term)

(9) $$Y_{v}=X_{h}\beta +\varepsilon$$

and test the null hypothesis that the coefficients β are zero: The multivariate test procedure hinges on the roots of the following expression of the null hypothesis (Chatfield and Collins, 1980)

(10) $$\begin{array}{l} \left|H-\theta R\right|=0\\ H=TT^{T}\\ R=(Y_{v}-T){\left(Y_{v}-T\right)}^{T}\\ T=X_{h}(X_{h}^{+}Y_{v})\text{.} \end{array}$$

The covariance explained by the least squares prediction *T* is *H*, whilst the unexplained covariance is *R*. In the simplest case of a one dimensional response, one can see that the single eigenvalue reduces to *θ* = *H*/*R*, which is the basis of the univariate F statistic. The more general multivariate statistic (Wilk's lambda) can be expressed as a function of the eigenvalues of R^− 1^H

(11) $$\begin{array}{l} \Lambda =\prod_{i=1}^{s}\frac{1}{1+\theta_{i}}:s=\min(v,h)\\ h=rank(X_{h})\\ r=n-v-h \end{array}$$

where *θ*_*i*_ are the ordered eigenvalues of *R*^− 1^*H* (in Eq. (10)). Wilk's lambda is effectively a (marginal) likelihood ratio test of the alternative hypothesis or model, specified by the contrast and the null hypothesis under Gaussian assumptions about the errors. For large *n* the log-likelihood ratio has a scaled Chi-squared distribution, with *v* × *h* degrees of freedom, where *r* (= n − v − h) is the degrees of freedom of the residuals (Chatfield and Collins, 1980)

(12) $$-\left(r-\frac{v-h+1}{2}\right)\ln\Lambda \sim \chi^{2}(vh)\text{.}$$

For *h* = 1, this statistic is equivalent to Hotelling's T^2^ and Roy's Maximum Root (as there is only one root). This concludes our description of how to form mass-multivariate SPMs. We now consider how to control the false positive rates when performing a massive number of multivariate tests over sources.

### Controlling family-wise error

**Fig. 1:**
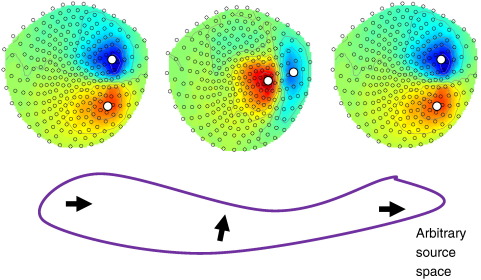
Example of the extremal heuristic. Three dipoles in an arbitary source space are considered. If this space is small in relation to the distance from the sensors then the left-most and right-most sources will have essentially identical lead fields, as they share the same orientation. Whereas the central source is distinct. The extremal heuristic in this case will be two (as there are only 2 unique extremal pairs) even though there are three sources in the space (and, in the random field context, all neighbours will have uncorrelated residuals).

In a mass-multivariate (SPM) setting we can finesse the inherent multiple comparisons problem using a Bonferroni correction for the effective number of independent sources within the source space, *ρ*. For readers familiar with random field theory in SPM, this would be like performing a Bonferroni correction for the number of resolution elements (or resels). Put simply, for a nominal family wise error rate of *α* the chi-squared statistic in Eq. (12) should exceed a threshold associated with a p-value of *α*/*ρ*. Clearly, this rests on knowing the number of independent sources: In this work, we examine three possible choices for *ρ*: the number of sensors, the number of sources, or the number of unique extremal pairs. The number of sensors is the number of independent measurements one begins with so it is an intuitive estimate of the number of independent sources. The total number of sources is an obvious overestimate, due to the inherent smoothness of source reconstructions. The extremal-pairs heuristic captures this redundancy and is based on the knowledge that, for an axial gradiometer or magnetometer system (see Discussion), the magnetic field due to a current dipole is well characterised by a function with a single maximum and minimum over space (Sarvas, 1987). This means that nearby dipolar sources with similar orientations (even those on either side of a sulcus) will be manifest in sensor space as (near) single entities. The number of unique extrema can be evaluated as follows: If **L** represents the *p* × *m* lead-field matrix (where each lead field is a row), then construct a new matrix with p rows and two columns containing the sensor indices of the maximum and minimum in each row; these correspond to the field map peaks for any lead field. The estimate of the number of independent elements *ρ* is then given by the number of unique rows (regardless of order of maxima and minima), which will (usually) be substantially less than the number of sources. A simple example of the extremal heuristic is shown in Fig. 1. Here three dipoles within a small, arbitrarily defined source space are considered. Since the left and the right sources are identically orientated, and they are separated by a distance that is small relative to the distance to the sensors, they exhibit essentially identical lead fields. The central source is distinct due to its different orientation. Since the extrema for the left and right sources fall at the same sensors, there are only 2 unique extrema pairs (hence 2 estimated spatial degrees of freedom).

### Testing the dimensionality of the alternative hypothesis

For the majority of this paper, the effects of interest X_h_ under test have rank *h* = 1. However, in the final example below, we show how the methodology naturally extends to *h* > 1. In these cases, tests for the dimensionality of the alternative hypothesis are based on a generalisation of Eq. (12)

(13) $$\left(r-\frac{v-h+1}{2}\right)\text{ln}\prod_{i=1+d}^{s}(1+0_{i})∼\chi^{2}((v-d)(h-d))$$

where *d* = 0 is equivalent to Eq. (12). That is, at each location deemed significant at a corrected level, we make a second test to determine the dimensionality of the alternative hypothesis by testing the significance of Eq. (13) for progressively increasing values of d. This tells us how many linear mixtures of X_h_ are significant; i.e., the dimensionality of the mapping from explanatory variables in X_h_ to the multivariate response variables at each source. Clearly, in the limiting case of one explanatory variable (*h* = 1) this dimensionality is only one or zero and *d* = *s* − *1* = 0. This is Hotelling's T-squared test, when Wilk's Lambda reduces to Roy's maximum root.

### Multivariate sensor test and mass-univariate source tests

In the current framework, a sensor level multivariate test can be implemented by replacing W in Eq. (7) with the identity matrix. This means we have a single multivariate sensor level test, in which there is no need for a Bonferroni correction. However, there is a correction for the implicit multiplicity of tests in source space, because the response over sensors is *m*-variate. Crucially, the philosophy of MVS means that the same (uncorrected) Chi-squared threshold can be applied to the corresponding source level mass-univariate (*k* = 1) Chi-squared statistics to control false positives; where the multivariate and mass-univariate Chi-squared statistics are obtained from the same data and design matrix. Note that this MVS threshold is formally distinct and (as we will see below) much more conservative than a Bonferroni correction for the number of sensors.

### Simulations

The measurement system configuration (gradiometer locations, head position *etc.*) for the simulated data was based on a real recording session using a 275 sensor CTF Omega system (with one sensor missing) with a sampling rate of 600 Hz. Unless otherwise stated, simulated data consisted of 200 trials and 274 MEG sensors. The sensor data were Gaussian noise of 10 fT/sqrt (Hz), over a bandwidth of 80 Hz. A single sphere head model was used to compute the lead fields. A dipolar source at the approximate location of the auditory cortex was driven at 40 Hz for a period of 200 ms to simulate signal. Source activity was reconstructed on grids of varying densities (5 mm, 10 mm, and 20 mm). We also reconstructed minimum norm estimates, using source spaces and orientations generated from a canonical brain mesh with 5124 vertices (Mattout et al., 2007). We used three different regions of interest to search for significant responses. These were based on the AAL atlas system (Tzourio-Mazoyer et al., 2002); the whole brain, the occipital lobe (with fusiform, lingual and calcarine included) and left Heschl's gyrus. These constitute the different source spaces we considered.

For each simulation we typically generated 100 SPMs of Chi-squared statistics using Eq. (12). In each run the rows of the design matrix were randomly permuted in order to create a null (permutation) distribution of the maximum Chi square values (across each ROI). This null distribution provides thresholds that control family wise error rates over the ROI, as in conventional statistical non-parametric mapping. We split our 400 permutations into four runs to get some measure of variability in the chi square threshold (minimum to maximum values reported in the error bars in subsequent figures). From this empirical null distribution it was possible to compare the permutation-based (corrected) significance level of a given Chi-squared statistic with that predicted analytically by Eq. (12). Note that as the ground truth is unknown, the permutation-based significance is our best estimate of the truth, however this is not known precisely (for 400 realisations and a nominal specificity of α = 0.05 one would expect a 95% confidence interval ranging from α = 0.03 to 0.076).

## Results

**Fig. 2:**
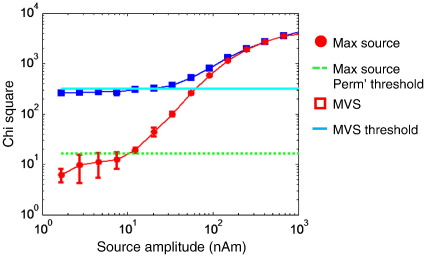
Comparison of sensor and maximum source level *χ*^2^ statistics from a ROI containing a single dipolar source of increasing amplitude: The simulated source was present in 500 of 1000 trials and comprised a sinusoid driven at 40 Hz for 200 ms. Source and sensor level tests are both based on the amplitude of the real Fourier component in the 40 Hz bin, comparing the active to passive trials. Blue squares show the multivariate sensor (MVS) level test statistic alongside the analytic sensor level threshold (cyan solid) for this test (for 1 feature, and 274 sensors this statistic has a *χ*^2^ (274–1) null distribution). The red circles show the maximum *χ*^2^ value from across the source space (1 cm grid) and the source level threshold (green dotted) shows the corrected p = 0.05 threshold level for the maximum *χ*^2^ statistic based on null distribution from 400 permutations. As all these tests consider a single data-feature, the source level *χ*^2^ test is equivalent to an F test and the sensory level multivariate *χ*^2^ is equivalent to the Roy's maximum root or Hotelling's T squared test. Note that the sensor level threshold (cyan solid) is an upper bound on the mass-univariate threshold (green dotted). Importantly, the source level statistic crosses the source level permutation threshold long before it crosses the threshold based on the multivariate test. That is, the multivariate sensor level threshold is a valid but rather conservative threshold for mass-univariate source level tests.

Fig. 2 shows the correspondence between multivariate and mass-univariate source space statistics motivating MVS (Carbonell et al., 2004). Sensor level multivariate tests (*k* = 1) were implemented by replacing **W** in Eq. (7) with the identity matrix. That is, tests were applied to *m* sensor data at a single frequency bin (the frequency of the simulated signal). It is not possible to apply a classical multivariate test when the number of features (channels in this case) exceeds the number of trials. A sensor level test like MVS therefore requires a large number of trials: For our first simulation (with 275 channels) we simulated 1000 trials. For an increasing source strength, the maximum source space Chi-squared statistic (red circles) increases monotonically and is bounded by the sensor level multivariate Chi-squared statistic (blue squares). Importantly, the MVS threshold (solid cyan) is a valid threshold for the source level test (and an exact threshold for the sensor level test); but it is also rather conservative (by approximately one order of magnitude) as compared to the source level permutation threshold (green line). This is because the MVS threshold is based on a correction for all possible linear sensor combinations, whereas the MEG source space comprises a finite number of sensor combinations (or lead fields), which are constrained by the Biot–Savart law to vary smoothly. In short, although the MVS thresholding procedure is valid is it very inefficient.

**Fig. 3:**
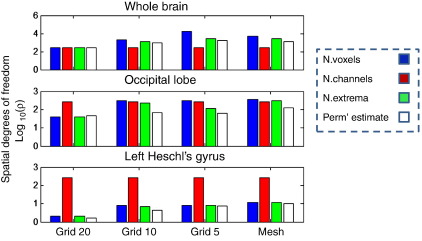
Estimates of the number of independent sources ρ based on the original number of sources (blue bars), the sensor count (red), and number of unique extrema (green) for three different regions of interest. Also shown (white bars) are the effective number of sources (or ideal ρ) estimated based on the Bonferroni correction required to give the desired family wise error rate in the univariate evoked response case (see next figure). Note the ordinates are logarithmic so a difference of 1.0 is equivalent to an order of magnitude. The source spaces considered here used different grid densities, where 5, 10, and 20 refer to regular lattice source spaces of 5, 10, and 20 mm spacings; mesh refers to a canonical cortical mesh of 5124 vertices. Note that the best predictors of ideal ρ are the total number of sources (blue) and the number of unique extrema (green), and for small (resp. coarse) source spaces these two metrics are approximately the same, but as the source space becomes larger (resp. finer) the extremal measure increases relatively slowly.

We next evaluated analytic source level thresholds based on Bonferroni corrections, in relation to the source level permutation threshold for null data. The analytic threshold requires a Bonferroni correction for the effective number of independent sources. We examined three possible estimates of this number: The number of sensors, which bounds the degrees of freedom one begins with; the number of sources, which is also an upper bound and the number of unique extrema, which is an approximation to the number of unique dipolar lead fields. Fig. 3 shows the total (blue) and the extremal estimate (green-bars) of *ρ* for different source spaces and regions of interest. For completeness, the number of sensors is shown in red. The extremal-heuristic is about the same as the number of sources for small regions of interest but increases slowly, relative to the total number of sources, with the size of the ROI and source space density (as many sources share the same lead fields). The permutation-based number of independent sources (white bars) was estimated as the level of Bonferroni correction required to make the Chi-squared distribution in Eq. (12) produce the permutation threshold (for a specificity of p = 0.05 corrected). It can be seen that the permutation and extremal heuristic numbers are in reasonable agreement, over the search spaces considered.

**Fig. 4:**
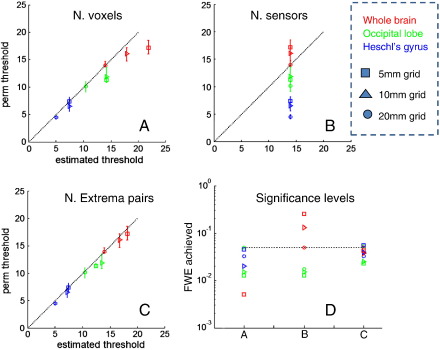
A comparison of analytical thresholds for a mass-univariate test based on three different estimates of the number of independent sources for a family wise error rate of 5%. The three estimates are the total number of sources (A), the number of sensors (B) and the number of unique extremal pairs (C). Different symbols represent different grid spacing (circles, 20 mm, triangles 10 mm, and squares 5 mm) and different colours represent different regions of interest (red whole brain, green occipital lobe, and blue Heschl's gyrus). The dotted lines show the ideal (exact) match between permutation and analytical thresholds, and points below this line indicate that analytical thresholds give conservative (larger) thresholds than required by permutation testing. The false positive rate (assuming that all sources are independent) is, as one would expect, always conservative but becomes more accurate as the number of sources decreases. The assumption that there are as many independent sources as there are sensors (B) gives inexact thresholds, which are generally conservative for smaller regions of interest (blue) and too liberal for larger ROIs (red). In panel C, the number of independent sources is based on the unique extremal heuristic, which provides efficient thresholds across all grid spacing and regions of interest. Panel D shows the actual and desired (dotted) error rates for each sort of threshold reported in panels A, B and C.

Fig. 4 shows the analytic source level thresholds based on each of these choices for *ρ* in relation to the permutation thresholds for three regions of interest (red — whole brain, green — occipital lobe, and blue — Heschl's gyrus) and three different reconstruction source spaces (circles — 20 mm, triangles — 10 mm, and squares — 5 mm). The ordinates show the permutation threshold (for p = 0.05 corrected), and the abscissa show analytic Chi-squared thresholds based on the different choices of *ρ*(panels A, B and C). Points below the dotted line mean the analytic estimates are too conservative. Fig. 4D shows the false positive rates corresponding to the desired five percent threshold estimates for each estimate of *ρ*. As one might expect, the number of sources (Fig. 4A) is always conservative, consistently over estimating the effective number of independent sources (as many adjacent dipoles share the same lead fields). As the region of interest shrinks, this threshold becomes closer to optimal (in the limit it is correct for one source). The number of sensors (Fig. 4B) is generally a poor predictor of the permutation threshold, providing too liberal a correction for high density source spaces (red symbols) and is too conservative for others (note that the analytic threshold is the same for all ROI and source spaces because the number of sensors is fixed). By contrast, the heuristic based on unique extrema produces mildly conservative but close to optimal control, across a range of source spaces and regions of interest (varying by 3 orders of magnitude in the number of sources). In panel D, one can see that the false positive rates using this threshold are mildly conservative in relation to the desired rate of five percent. Note that the MVS threshold of 312.5 based on *χ*^2^(*m − 1* = 273) is vastly more conservative than any of the thresholds on source space statistics considered here.

**Fig. 5:**
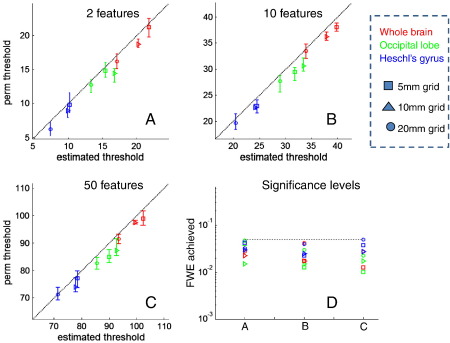
A comparison of mass-multivariate thresholds using the unique extrema for v = 2 (A), 10 (B), and 50(C) data features. This figure uses the same notation for grid spacing (symbols) and ROIs (colours) as in Fig. 3. Panel D shows the actual and desired (dotted) error rates for each of the tests reported in panels A, B, and C. This estimate of the number of unique sources furnishes good, although mildly conservative, mass-multivariate thresholds.

In Fig. 5, we use the unique extrema count to approximate the number of independent sources and compute Bonferroni corrected thresholds for the different source spaces and regions of interest. Here, we consider mass-multivariate tests involving more than one feature at each source location. Panels A, B, and C correspond to tests on v = 2, 10, and 50 features per source. Panel D shows the achieved false positive rate. The heuristic gives mildly conservative thresholds, in relation to the permutation threshold, across tests of all dimensions. Again, note that these thresholds are orders of magnitude below the MVS threshold, which would be (given sufficient numbers of trials): *χ*^2^(2 × 274 − 1) = 602.5, *χ*^2^ (10 × 274 − 1) = 28,619 and *χ*^2^ (50 × 274 − 1) = 13,972.

**Fig. 6:**
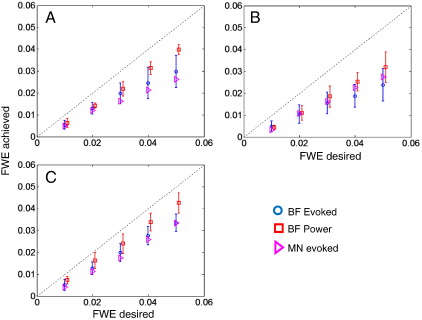
The desired vs. actual (permutation) false positive rates, collapsing results from all feature numbers (1, 2, 5, 10, 20, and 50) and grid spacing for tests on evoked (blue circles) and induced (red squares) responses for whole brain (A), occipital (B) and Heschl's gyrus (C) ROIs. The error bars show the standard deviation. Note that the tests of power seem to be less conservative than those of evoked responses. The purple triangles are the false positive rates when applying the same heuristic to the mass-multivariate minimum norm scheme (Soto et al., 2009) reconstructed on the canonical mesh.

We examined permutation and analytical thresholds for all source space densities, ROIs, and a range of (1, 2, 5, 10, 20, and 50) spectral data features, for induced (power) and evoked responses. To see if the heuristic failed for particular source spaces or regions of interest, we tested for differences amongst the actual (permutation) corrected p-values obtained with an analytic threshold of p = 0.05 (corrected). Collapsing across all features (for the whole brain ROI), we found no effect of source space (F(2,15) = 1.4, p = 0.278). Collapsing across grid spacing, we found no effect of features (F(5,12) = 1.33, p = 0.315). There was an effect of ROI (using 5 mm spacing, collapsing over features) (F(2,15) = 3.74, p < 0.0481). The mean corrected p-values here were p = 0.0292, 0.0212, and 0.0379, for whole brain, occipital, and Heschl's gyri, respectively. There was also a significant difference depending on whether one tested for power or evoked response (F(1,10) = 5.98, p < 0.0346) (collapsing over features for finest grid and whole brain), corrected p-values were p = 0.0292 and 0.0442 for evoked and power tests respectively. Fig. 6 shows the desired and actual false positive rates over the standard beamformer grids for the three regions of interest (A whole brain, B occipital lobe, and C left Heschl's gyrus) averaged over all data feature numbers and grid spacing. False positive rates for induced responses are shown as red squares and for evoked as blue circles. The purple triangles show tests on evoked response for a minimum norm solution (Soto et al., 2009) on a canonical mesh (again collapsed across all features). These results suggest that the validity of the extremal-heuristic is not restricted to specific reconstructions or source space topology. Note from Fig. 3 that the number of original sources varies by 3 orders of magnitude across the three regions of interest but that the false positive rates remain controlled using analytic thresholds (compare panels A and C in Fig. 6).

**Fig. 7:**
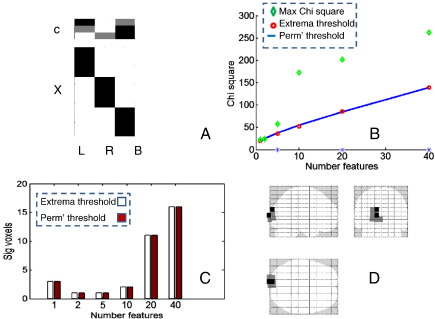
Application of the scheme to test for differences between gamma spectra due to two gratings of different orientations and a pre-stimulus baseline period (Duncan et al., 2010). Panel A shows the design (lower) and contrast matrices (top); there are 3 conditions (left oblique, right oblique, and blank) and we wish to test for differences between all three conditions (color scale is black, midgrey, and white for − 1, 0,+1 respectively). Panel B shows the maximum source level statistic (green diamonds), the analytical threshold based on the unique extremal heuristic (blue solid) and the permutation thresholds for the volume (red circles) for different numbers of spectral features (i.e., principal eigenmodes). Note that the analytical and empirical thresholds accord well, and panel C shows the agreement between the numbers of voxels deemed significant using both thresholds (permutation solid). Stars on the abscissa (in B) indicate that the dimensionality of the alternate hypothesis was more than one. D shows the glass brain image for the (FWE corrected) significant voxels (grey) for mass-multivariate tests on 20 spectral features (eigenmodes). Those voxels where 2 dimensions were required to explain the alternative hypothesis are shown in darker grey; that is, at these sources the differences between gamma spectra cannot simply be explained by a scaled difference along one dimension (for example left oblique > right oblique > baseline) but must be due to distinct spectral responses to left and right oblique gratings.

In Fig. 7, we apply the multivariate beamformer test to a single subject from the study of Duncan et al. (2010). Stimuli were stationary square wave gratings presented either to the lower right or left of fixation (1.5° in extent, 3 cpd, 80% contrast) obliquely oriented at either 45°(right) or 45° (left oblique) to vertical (Fig. 1A). Randomly intermixed (left or right oblique) stimuli were presented for 2.5 s and followed by a 2.5-s period of a uniform field of the same mean luminance (baseline period). Subjects were simply asked to look at the fixation spot. The experiment comprised two (left and right visual fields) 800-s runs, each of which contained 80 presentations of each stimulus type. The authors were interested in whether the standing gamma oscillation (300 ms–2.5 s) could be used to identify the orientation of the grating. The authors first identified a region of interest by looking for changes from baseline using a mass-univariate test. At this location they went on to show that different grating orientations give rise to subtly different spectra using a single multivariate test. In this example, we begin with the alternate hypothesis of one or more differences amongst the two grating and pre-stimulus baseline conditions and examined different data-features (increasing number of spectral eigenvectors or modes), whilst testing (and correcting) over the whole brain. Fig. 7A shows the design and contrast matrices specifying the mass-multivariate tests. Fig. 7B shows the permutation (red circle) and heuristic (blue line) thresholds alongside the maximal Chi-squared statistic over source space (green diamonds). Panel C shows that the identical number of voxels is deemed significant for both the permutation and analytic thresholds in this real-world example. Fig. 7D shows the same significant voxels plotted in the glass brain as grey dots. The test statistic here is no longer equivalent to Roy's maximum root or Hotelling's T square as *h* = 2 and there are two degrees of freedom in this design (i.e., two potential differences amongst the three conditions). The validity of this treatment of Wilk's lambda is important (see Discussion). Duncan et al. (2010) replaced the power spectra due to the different stimuli by their rank transforms in order to show that the spectra did not differ simply in amplitude (but rather in shape). We can now address this issue more directly and ask if the differences amongst the three stimulus conditions (left oblique, right oblique and baseline) fall on a single response dimension (as would be the case if left oblique spectrum gave rise to displacement from baseline equivalent to a scaled right-oblique spectrum) or whether this difference is two-dimensional; in which case, one would need at least two orthogonal differences to describe the change in the spectrum, showing that the three responses are distinct. This rests on testing for the dimensionality of the responses using Eq. (13).

In this case the response has at most two dimensions (rank(X_h_) = 2) so we can test if the response spans more than one dimension by using Eq. (13) with d = 1. When we applied this test at all significant voxels from the first pass (grey dots in Fig. 7D) we found two-dimensional effects to be significant in visual cortex (black dots: Fig. 7D). Note that, if required, the directions of these responses could be extracted from the canonical vectors of *R*^− 1^*H* (see Soto et al., 2009). In turn, these can be multiplied by c to characterise the effects in terms of the contrasts.

## Discussion

We began by looking at how well the MVS method (Carbonell et al., 2004) approximated mass-univariate source level permutation thresholds. Although theoretically exact, this method provides a conservative upper bound for thresholding source space SPMs, as the lead fields comprise a small subset of all possible linear sensor combinations. We went on to show that source level permutation thresholds are well approximated using a simple heuristic based on the number of unique extrema of the lead fields. This heuristic holds across mass-univariate and mass-multivariate tests on different source spaces and regions of interest. The incorporation of the more general Wilk's lambda means that one can test for effects which exist in more than one response dimension and indeed produce SPMs of such dimensionality (see Fig. 7).

We note that although multivariate sensor level tests have been shown to be extremely sensitive (Carbonell et al., 2004; Friston et al., 1996; Fuentemilla et al., 2010), they have no localising power. Furthermore, controlling source level tests with sensor level thresholds can be very insensitive. The relative sensitivity of our Bonferroni approach derives from knowing the linear sensor combinations a-priori. Mass-multivariate source level tests are efficient in their use of degrees of freedom, as the linear-sensor combinations (entailed by the form of lead fields) do not have to be estimated. That is, for a source level test there is a cost of one degree of freedom per extra temporal feature whereas for a sensor level test this is multiplied by the number of effective sensors (n × v). This means we were able to perform tests on up to 50 spectral features per source element with only 200 observations (trials). To make even a single feature sensor level test on this number of trials one would need to reduce the number of sensors to around 70 (see Friston et al., 1996). We should note also that the accuracy of the estimate of W in Eq. (3) also depends on the number of trials (Brookes et al., 2008).

It is of course also possible to compute the MVS threshold through permutation rather than using the analytical estimate. The advantage of the permutation method is that it makes no assumptions about the underlying data distribution. For the data we looked at (both real and simulated), we found the two estimates to be in very close agreement. However, further work in this area would allow one to state whether the basic assumptions behind the multivariate test (such as multivariate normality) are valid for typical MEG data.

In our next study, we will compare the extremal heuristic with corrections for multiple comparisons based on random field theory. The attractive property of random field theory is that it allows one to correct over regions of inhomogeneous smoothness, based on estimates of the local spatial derivative in the 3D MEG source space(Barnes and Hillebrand, 2003; Pantazis et al., 2005). The extremal heuristic operates in *m*-sensor space, which has the advantage that it is sensitive to non-local covariance structure (for example similarly orientated sources separated by a source of a different orientation) to which the local random field operator is blind. That is, by operating in sensor space, one circumvents the need for local smoothness estimators. On that note the main assumption here is that the dominant noise source is at the sensor level (i.e. sensor white noise), rather than the source level (physiological noise). Empirically, this seems to be a reasonable assumption (Fig. 7); however, if there is considerable image smoothness at the source level (due to spatially correlated physiological noise processes) then this heuristic may fail (whereas RFT will remain unaffected). This is clearly an area where more work is required but initial comparison between univariate stationary random field theory and this heuristic suggests that they have comparable sensitivity (Supplementary Fig. 2). A key advantage over random field theory is that our heuristic-based Bonferroni correction can easily accommodate multivariate test statistics; for example, besides being rather complex to derive, current threshold estimates based on random field theory for Wilk's Lambda are conservative (by a factor of 4) with respect to random field theory based on Roy's maximum root (Carbonell et al., 2009) (for the same multivariate test). Importantly the heuristic we propose is not limited to Wilk's lambda but could be applied to any parametric or non-parametric test (as the heuristic simply estimates the effective number of tests). Although by the Neyman–Pearson lemma the likelihood ratio test used here is the most efficient of all statistics and is a special case of a Bayes factor (Friston et al., 2007: Appendix 1).

Our heuristic Bonferroni approximation holds for both beamformer and minimum norm solutions and we expect it to hold for the majority of MEG inversion algorithms, as it is based on the dipolar lead field. Similarly the topology of the source space was not a factor, as the estimate of the number of independent sources is made in sensor space and not source level. One straightforward application might be to use solutions, such as MSP (Friston et al., 2008b), where source space is already partitioned. In this case, the number of unique elements could correspond to the number of patches which have distinct extrema, and indeed such a heuristic could be used to remove redundancy between patches prior to source reconstruction and testing.

In this study, we have concentrated on within subject statistics, although the heuristic could be applied to inversion schemes in which canonical sensor and cortical bases are used to pool data from multiple subjects (Litvak and Friston, 2008). Computationally the permutation tests are expensive and we have only been able to validate our findings up to a grid sampling of 5 mm (approximately 4 days per curve on a state of the art PC). We do not expect denser source spaces to pose a problem as the increasing density will make only a marginal difference to the number of unique extrema (due to the intrinsic smoothness of source space imposed by the Biot–Savart law (Sarvas, 1987). One issue, not addressed here, is the sampling of the sensor space. We found that the heuristic does not hold (becomes too liberal) when the MEG sensor number is small (< 50 sensors); i.e. the source space is undersampled and there is an aliasing problem, in which a non trivial number of different dipolar fields appear as maxima on the same MEG sensors. Conversely, the generally conservative analytical estimates observed maybe due to an oversampling of the sensor space. One can imagine a situation where one has sensors at millimetre spacing: tiny numerical differences in the lead fields between these locations will give rise to an inflated number of unique extrema, leading to yet more conservative tests. One solution could be the re-sampling of sensor space to the optimum close to the Nyquist frequency of doubling the maximum spatial frequency due to any lead field originating from the source space. We are also currently working on further heuristics based on the eigen-structure of the lead fields.

The simulations here assumed an axial gradiometer system (in which case a dipolar source has two distinct extrema). We were interested to see if the same extremal heuristic could be applied to systems consisting of a mixture of planar gradiometers and magnetometers. To address this, we re-ran our simulations using a Neuromag vector-view configuration. We found that the extremal heuristic, based purely on the extrema in the magnetometer lead fields, did a good job of predicting false positive rates for source reconstructions using the full sensor set of gradiometers and magnetometers (please see Supplementary Fig. 1). Similarly, in this simulation we used a single sphere forward model but the same qualitative findings hold for multi-sphere (Huang et al., 1999) and single shell (Nolte, 2003) models (data not shown).

In contrast to Soto et al. (2009) we have chosen to make a decomposition of the spectrum into those components with maximum variance, rather than choose specific bands a priori. One problem with both approaches is to make some principled decision as to how many (and which) spectral features (resp. bands) should be used. Indeed one could also apply exactly the same algorithm to the time-frequency wavelet decomposition at each source location. One method, which could be used to make this judgment, either at, or across sources, would be multivariate Bayes (Friston et al., 2008a). At present this is computationally challenging but could be a promising direction of future research. Another issue we have not investigated here is the grouping of similar canonical vectors across source space; this would give some indication as to which elements respond similarly to the stimulus and might even provide some information to help distinguish between cortical areas.

We have introduced mass-multivariate SPM in a beamformer framework, which is very similar to the approach described by Soto et al. (2009) for the minimum norm solution. We expect there are to be considerable advantages to our approach in the case of the beamformer. This is because beamforming allows the use of wide covariance windows (and robust covariance estimates that ensue (Brookes et al., 2008). It also provides an objective feature selection (through the eigen decomposition) within this window, removing the need for arbitrary choices of frequency bands of interest, and the inherent multiple comparison problems. The applications of mass-multivariate tests to M/EEG data are manifold as they allow one to exploit the rich temporal structure within the data. We expect large benefits over univariate tests when a task is characterised by power decreases in some frequency bands (or time windows) and increases in others (Singh et al., 2002). As suggested by Soto et al. (2010) the design matrix could itself be a measurement (of power or phase at a particular location) and one could begin to look for dependencies between different regions of time-frequency space across the cortex (Canolty et al., 2006; Schoffelen and Gross, 2009).
