# Supplementary material for: Controlling false positive rates in mass-multivariate tests for electromagnetic responses
Source: Neuroimage. 2011 Jun 1;56(3):1072–81. doi: 10.1016/j.neuroimage.2011.02.072 (PMC3092987; doi:10.1016/j.neuroimage.2011.02.072)
Supplement: Supplementary file 1 — Supplementary material. [file mmc1.ppt]

## Slide 1
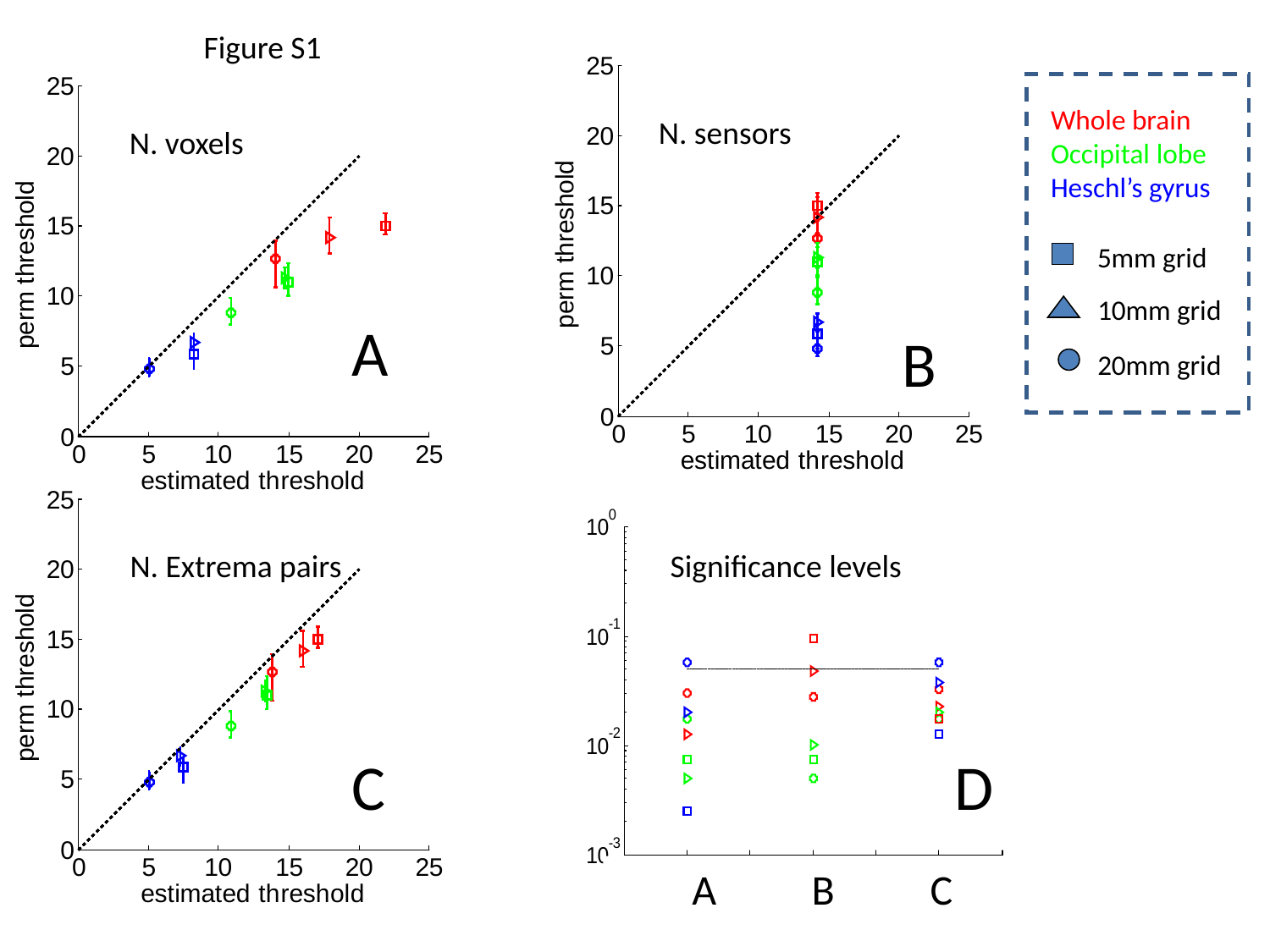

Figure S1
Whole brain
Occipital lobe
Heschl’s gyrus
N. sensors
N. voxels
5mm grid
10mm grid
A
B
20mm grid
N. Extrema pairs
Significance levels
C
D
 A B C

## Slide 2
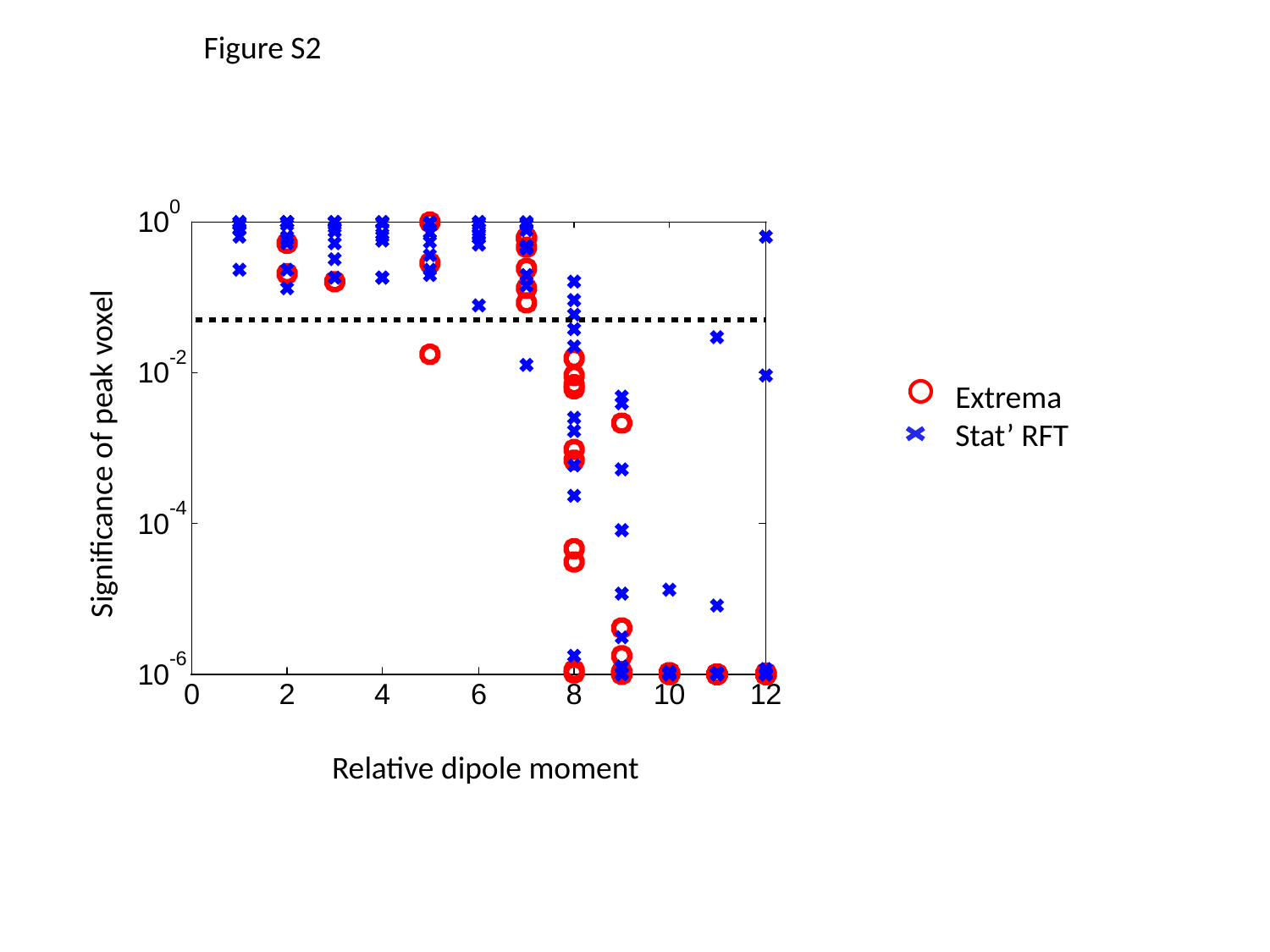

Figure S2
Extrema
Stat’ RFT
Significance of peak voxel
Relative dipole moment
